# AI Moral Enhancement: Upgrading the Socio-Technical System of Moral Engagement

**DOI:** 10.1007/s11948-023-00428-2

**Published:** 2023-03-23

**Authors:** Richard Volkman, Katleen Gabriels

**Affiliations:** 1 Department of Philosophy, Southern Connecticut State University, 501 Crescent Street, New Haven, CT 06515 USA; 2 Department of Philosophy, Maastricht University, FASoS, Grote Gracht 90-92, 6211 PG Maastricht, The Netherlands

**Keywords:** Moral enhancement, Artificial intelligence, Artificial moral agent, AI socratic interlocutor

## Abstract

Several proposals for moral enhancement would use AI to augment (auxiliary enhancement) or even supplant (exhaustive enhancement) human moral reasoning or judgment. Exhaustive enhancement proposals conceive AI as some self-contained oracle whose superiority to our own moral abilities is manifest in its ability to reliably deliver the ‘right’ answers to all our moral problems. We think this is a mistaken way to frame the project, as it presumes that we already know many things that we are still in the process of working out, and reflecting on this fact reveals challenges even for auxiliary proposals that eschew the oracular approach. We argue there is nonetheless a substantial role that ‘AI mentors’ could play in our moral education and training. Expanding on the idea of an AI Socratic Interlocutor, we propose a modular system of multiple AI interlocutors with their own distinct points of view reflecting their training in a diversity of concrete wisdom traditions. This approach minimizes any risk of moral disengagement, while the existence of multiple modules from a diversity of traditions ensures pluralism is preserved. We conclude with reflections on how all this relates to the broader notion of moral transcendence implicated in the project of AI moral enhancement, contending it is precisely the whole concrete socio-technical system of moral engagement that we need to model if we are to pursue moral enhancement.

## Introduction

An ongoing conversation in the philosophical literature explores the use of artificial intelligence (AI) as a means of moral enhancement (see e.g., Savulescu & Maslen, 2015). For instance, Giubilini and Savulescu (2018, p. 170) consider humans as “suboptimal information processors”. Due to stress and time constraints, we often fail to consider all the relevant information necessary to arrive at well-reasoned moral judgment, we lack consistency, and we are prone to bias. To make up for these shortcomings, they propose the assistance of an artificial moral advisor. Unlike humans, AI can collect, analyze, and process an enormous amount of data effortlessly, which they believe would lead to more informed and better moral judgments.

Our contribution explores several proposals for moral enhancement, ranging from those that use AI to supplement (auxiliary) human moral reasoning or judgment to those that would even supplant it (exhaustive). We find existing proposals typically abstract away from the concrete mechanisms of moral improvement known to date—namely, our current socio-technical system of moral enhancement comprised of people connected by information technology engaged in moral discourse. At the extremes, these proposals (e.g., Dietrich, 2001) conceive the AI as some self-contained oracle whose superiority to our own moral abilities is manifest in its ability to reliably deliver the ‘right’ answers to our moral problems. We think this is a mistaken way to frame the project; it presumes that we already know many things that we do not know already and may never know. It is at least premature to consider developing an AI system to do our moral thinking for us.

Nonetheless, we agree with many proposals that suggest there is still a substantial role for AI to play as tools of auxiliary moral enhancement. Specifically, interactive ‘*AI mentors’* could outshine books and other media in moral training. We enlarge on the idea of an AI Socratic Interlocutor that can carry on an adequate conversation in some or another specific wisdom tradition, operating like an interactive book. An ‘AI mentor’ could contribute to training, especially in wisdom traditions such as Stoicism, Buddhism, Aristotelianism, and others where rigorous and ongoing training to develop practical wisdom is at the very core of moral improvement. Such a technology is a more modest gesture towards moral enhancement than the more transhumanist, exhaustive proposals. Rather than aspiring merely to give the ‘right’ answers, as an advisor might do, an AI mentor helps to cultivate the users’ own practical wisdom by offering guidance.

The paper unfolds in five parts. First, we present an overview of AI moral enhancement and its challenges, especially in its most ambitious forms. Next, we argue that more moderate proposals are still vulnerable to objections, especially relating to pluralism. This sets the stage for a discussion of an AI Socratic Interlocutor, as proposed in Lara and Deckers (2020) and extended in Lara (2021). While agreeing that their proposal points in the right direction, we argue that it still fails to model the system of moral engagement that has brought us where we are today—the socio-technical system of humans discussing and debating moral matters via communications technologies like books and speech. To remedy this, we propose a modular system of multiple AI interlocutors with their own distinct points of view reflecting their training in a diversity of concrete wisdom traditions. We conclude with reflections on how all this relates to the broader notion of moral transcendence implicated in the project of AI moral enhancement.

## Moral Enhancement is Not Just About the ‘Right’ Answers

Some proposals for technological moral enhancement (e.g., Dietrich, 2001) measure moral enhancement in terms of getting the ‘right’ answers, ultimately reducing morality to the output of some algorithm. We contend this is misguided. Beyond the right answers, our thinking about morality also matters. Relying on an AI to do our thinking for us not only neglects the cultivation of moral excellence but actively undermines it, exposing agents to risks of disengagement, atrophy of human faculties, and moral manipulation at the hands of the AI or its creators. All of this seems to be the very opposite of enhancement.

To appreciate the significance of these challenges, we need some understanding of how practical wisdom appears in humans. We do not deny that an AI might model human practical wisdom. Rather, we emphasize the dynamic, contested, and complicated nature of practical wisdom, such that AI moral enhancement cannot simply upgrade our socio-technical systems of moral education and inquiry by supplanting the human element in the system. We still need human judgments to discern whether the AI’s suggestions are pointing in the right direction, and human contributions to the information processing can be unpredictable and incremental, so AI moral enhancement will require an interface between AI and human that allows for the unfolding complexity of discourse relating to dynamic and contested notions. Any machine adequate to answering our questions will pose risks to our abilities to answer questions for ourselves, and our abilities to answer for ourselves are a necessary part of the still open-ended process we mean to enhance. While there is little agreement about how to define morality and how people arrive at moral judgments, there is good reason to think our faculties of moral judgment will become weaker if these faculties are not vigorously exercised, since moral judgment is an activity that involves a complex interplay of affective and cognitive faculties, and skillful activity generally requires practice and coaching (cf. Vallor, 2015). While only the most extreme proposals for AI moral enhancement explicitly treat the AI as an oracle delivering ‘right’ answers, a firm understanding of why that view is widely rejected helps to inform our critique of less ambitious proposals.

Setting aside philosophical prescriptions for the moment, results in empirical psychology reveal that we are astonishingly ignorant even of much of our own moral mental machinery. Moral psychologist Jonathan Haidt (2013) defines morality as learned sets of values, virtues, norms, practices, and innate psychological mechanisms that work together to regulate self-interest and enable cooperative societies. Haidt’s empirical study of how humans think about morality suggests our affective responses and intuitions primarily guide our moral reasoning. Thus, Haidt follows David Hume (1740): “Reason is, and ought only to be, the slave of the passions”. To illustrate, Haidt presented subjects with a taboo scenario about a brother and sister having sex with each other once. Some possible objections are covered in the scenario: the brother and sister use contraception and the sex takes place with mutual consent. Most respondents intuitively rejected the scenario and subsequently looked for arguments to support that intuition. Subjects often found themselves ‘dumbfounded’, since their Western Educated Industrial Rich Democratic cultures do not cultivate articulation of moral reasons based on certain affective foundations (such as sanctity, loyalty, and authority). Given more time to think about it and a substantiated argument, respondents may come to agree that the brother and sister can have sex once, but many cling to their initial intuitive response even after repeated failed attempts to articulate a rational justification. Intuitions and judgments can change in a conversation in which arguments are presented. So, reason may not be completely enslaved to our passions. However, Haidt’s main point in these ‘moral-dumbfounding’ experiments is to indicate how little we each already understand about our own moral perspectives, let alone about all of morality in the once-and-for-all manner supposed by moral enhancement as delivering ‘right’ answers.

Such accounts of the mechanisms of moral judgment complicate what it could mean to imagine an AI delivering ‘right’ answers to moral questions. Would a morally competent AI approve or disapprove of the proposed incest? If it is tracking what we care about in morality, should it not be as dumbfounded as we are? While maybe no one can confidently declare the right course of action in dumbfounding scenarios, there is a great difference between those who can articulate and defend their views and those who cannot, and that ability to carry on the moral conversation is an appropriate object of moral enhancement even if we admit no one already knows the ‘right’ answers. But that ability to participate in the conversation—plausibly the very mechanism of moral progress—is undermined insofar as we just do as some AI says. This risks fostering moral disengagement, a mechanism we use in everyday life to justify or excuse questionable behavior (Bandura, 2002), as when a CEO deflects from addressing the moral significance of a decision by appealing to narrowly economic considerations. Furthermore, it is widely agreed since Nuremberg that we should not just follow the orders of any human, and we should be no more comfortable just following the orders of an AI.

Many scholars, including some who would reject the idea of exhaustive AI moral enhancement, have nonetheless been attracted to AI moral enhancement on the grounds that a morally competent AI could outperform humans. Giubilini and Savulescu (2018) point to efficiency, speed, and consistency, among other things, to show AI can or will be better than humans. However, this eschews claiming to give the ‘right’ substantive answers in favor of claiming to know already the ‘right’ formal values to enhance. But are these criteria always preferable (see also Gabriels, 2021, pp. 52–54)? And do they, combined, automatically lead to moral enhancement? Of course, there are well-known situations where human nature can get in the way. AI might be, in principle, less vulnerable to in-group bias, responding to stimuli with unflustered equanimity and robotic consistency. Yet, it can be good not to be consistent, or at least not in such an inflexible way. Killing in self-defense violates the moral rule ‘do not kill' but warrants an ethical and legal evaluation unlike killing for gain. Evaluating deviations from a moral rule demands context, but it is extremely difficult to teach an AI to reliably discriminate between contexts.

Even legal judgments are not so unambiguously algorithmic as it might seem. Sometimes a violation of the law is ethically and even legally justified. When Rosa Parks refused to give up her seat on the bus to a white passenger in Alabama in 1955, she did something illegal. It was mandated by law that black people had to give up their seats to whites if all the seats reserved for them were occupied. Parks refused this, committing an illegal act, and refused to subsequently pay the fine imposed on her, a second illegal act. Both acts are ethically justifiable. They led to major breakthroughs for the American civil rights movement, fueled by anger and feelings of injustice. Having emotions may be essential to make society morally better. Having an AI that is consistent and compliant with existing norms and laws could thus jeopardize moral progress.

Likewise, speed and efficiency are not always better. There are surely contexts in which urgency demands a trade-off between speed and other values—including even accuracy. But even then, quality of output must be considered. Meanwhile, in a world of scarce resources (including time), efficiency matters. However, there are countless examples of AI making biased decisions or following an undesirable pattern because of overvaluing speed and efficiency.

So, it seems that getting an AI to deliver sound advice to humans will demand that it be able to speak to us in our own moral tongue, but it is not clear how to build moral intuition and nuanced discernment into technology. Rule-based AI seems to be ruled out from the start. People learn intuition and know-how through practice and experience (Dreyfus & Dreyfus, 1986), and human intuition cannot be straightforwardly formalized. Data-based AI suggests possibilities for AI to develop a kind of moral ‘intuition’ by having algorithms look for patterns in supplied data. Confronted with millions of moral scenarios, an algorithm analogous to DeepMind’s AlphaGo could 'play' through them (as a form of self-play) and learn from mistakes. Supposing we can reliably indicate mistakes to it, the AI will find patterns and thus develop a kind of ‘intuition’.

But even supposing moral intuition and nuanced judgment can be built into technology, we have yet to arrive at a proper moral judgment. A 'good' and convincing moral judgment goes beyond intuition; it is substantiated with arguments and goes further than a clear-cut yes (good) or no (bad). If someone judges a specific action to be wrong, they must be able to explain *why*. Only thus can we estimate to what extent the judgment is arbitrary or subject to prejudice. So, AI will also have to learn to argue. In the legal domain, research is ongoing to explore how AI can be used to assist lawyers in evaluating legal argumentation (e.g., see Walton, 2016), and interesting research is being done within Natural Language Processing (NLP).

While technical challenges remain, substantial progress is being made to overcome them. Even today, machines perform many sophisticated analyses as well or better than humans, for instance in the context of medical decision-making (e.g., Esteva et al., 2017), sometimes in ways human observers would not themselves anticipate. AlphaGo defeated world champion Lee Sedol in 2016 in the Chinese board game Go. By playing millions of matches, AlphaGo discovered strategies. During the game with Sedol, AlphaGo made a move experts initially thought must be a mistake. It turned out to be a brilliant move showing great insight. For a long time, it was thought you simply get out of algorithms what you put in, but AlphaGo surprised observers and made people play Go differently by trying out the new moves (Du Sautoy, 2019; see also Gabriels, 2021, p. 12).

So, perhaps a sufficiently advanced AI could learn the ‘moves’ of morality as well or better than any human, despite morality being a significantly more complicated ‘game’. Go is a 'zero-sum game': one side wins as much as the other side loses, so the net profit is zero. But there are also cooperative board games such as Hanabi and Diplomacy. DeepMind trained algorithms to play Diplomacy in a multi-agent environment by using reinforcement learning. In this approach, the system needs to discern which decision triggered the feedback in question, and what exactly needs to be reinforced or inhibited (Boden, 2016). Repeated tests show artificial agents performing better and better (Anthony et al., 2020). A research team at Meta developed an AI agent, called ‘Cicero’, that integrates a dialogue module and a strategic reasoning module, and reinforcement learning algorithms. Cicero achieved human-level performance in Diplomacy (see FAIR et al., 2022). Insofar as the coalitional thinking necessary for success in Diplomacy is a model of moral thinking, there is reason to expect an AI can eventually learn moral thinking.

However, it does not follow that it would be wise to let an AI do our moral thinking for us. Without humans doing the thinking for themselves, there would be no standard against which to measure the accuracy and reliability of the machines. We do not know whether the machine delivers the ‘right’ answers unless we already know the ‘right’ answers, which we do not. So, humans must remain as agents within the socio-technical system of moral enhancement comprised of people connected by information technology engaged in moral discourse, since the process is messy, unpredictable, complex, and contested along every dimension. This amplifies the dangers of disengagement noted above. If our own moral skills atrophy due to reliance on some moral technology, there is no mechanism to correct whatever errors and distortions might creep into the plausibly alien thinking of the machines. Even if the machines become full-blown moral agents in their own rights, they will not be human agents. Does that matter? This is among the contested moral questions we cannot answer confidently at present. Risks of disengagement only increase with greater AI competence inviting our greater reliance on its judgments rather than our own. This also goes for more modest proposals insofar as these too conceive enhancement as getting the ‘right’ answers. As most commenters in this space agree, getting machines to do our thinking for us is a bad idea.

## Exhaustive vs. Auxiliary AI Moral Enhancement

So far, we saw the most ambitious proposals expose us to risks of disengagement. To see why these concerns are not entirely remedied in less ambitious proposals, it is helpful to categorize proposals for AI moral enhancement as “exhaustive” or “auxiliary” (as proposed in Lara & Deckers, 2020). Exhaustive enhancement imagines machines morally superior to humans, such that just doing as the machine says constitutes moral improvement. Perhaps the clearest example of the exhaustive approach comes in Dietrich (2001), who contends machines will outperform us morally to such a degree that humans should (morally) choose our own extinction, handing the planet over to our morally superior non-human descendants, whom he calls “homo sapiens 2.0”. Certain transhumanist projects of moral enhancement advise that we integrate with and become the new species that replaces humans; morally enhanced post-humans will have transcended the various limitations defining humanity. But, as outlined above, some of our alleged shortcomings (e.g., emotions) seem to be the very mechanism of our evolved moral responses, leaving it unclear what morality means in their absence.

Auxiliary enhancement means to address these concerns, emphasizing the agent’s active participation in decision-making as a crucial aspect of the process of enhancement. On such approaches, an AI assistant is merely a tool to help the agent to clarify and keep track of moral commitments and contexts, while the ultimate decision-making rests with the agent who consults the AI as a resource. Savulescu and Maslen (2015) propose a weak moral AI whose parameters are tailored to match the moral commitments of the user, such that the AI can *assist* in moral decision-making. They contend, “an agent-tailored moral AI would not only preserve pluralism of moral values but would also enhance the agent’s autonomy by helping him to overcome his natural psychological limitations” (Savulescu & Maslen, 2015, p. 79). Giubilini and Savulescu (2018, p. 171, emphasis original) extend this general approach by framing the artificial moral advisor as a “*quasi*-relativistic version of the ‘ideal observer’ famously described by Roderick Firth”. This adds greater normative content to the proposal by grounding it in a relatively neutral conception of morality that still preserves pluralism. That is, it introduces minimal standards of evaluation beyond merely being consistent and well-informed as a framework that does not presume to determine all the ‘right’ answers in itself.

That greater normative content is welcome considering criticism from Klincewicz (2016, p. 177), in which neutral strategies to enhancement are challenged because “the moral AI would have to play not only an advisory or facilitative role, but also a normative one”. Merely making agents more consistent and thoughtful is not sufficient to produce moral enhancement, since users might input the wrong values from the start and become more consistently wrong as a result. This is an important point, but it should not be overstated. Neutral strategies operate from the presumption that one ought to be consistent and well-informed in making decisions. This is a purposely minimalistic normative commitment, but it is a mistake to suppose this minimalism means the system cannot help us to overcome certain moral shortcomings, unless one also assumes individuals are not merely bad at implementing their own visions of morality but are not even on the path. That assumption points back to the exhaustive project, since it suggests individuals not already on some path leading to moral improvement with greater clarity, consistency, and knowledge would just have to obey the machine. Nonetheless, Klincewicz proposes an auxiliary enhancement project building in more substantive normative commitments but aiming to persuade the user of moral truths rather than coercing or supplanting the user’s own decision-making.

The later proposal of Giubilini and Savulescu (2018) to frame the artificial moral advisor in terms of “ideal observer” theory similarly contributes normative content to the system while preserving substantial neutrality and pluralism, but this too suffers from relying on alleged moral knowledge we simply do not have. While many philosophers are convinced an ideal observer theory captures at a very abstract level what morality is all about, many more are not. Compared to alternative normative theories like consequentialism, deontology, and virtue ethics or to alternative meta-ethical theories like error-theory and moral realism, ideal observer theory remains a marginal player. Of course, pluralism itself is controversial, especially insofar as monist critiques associate it with some kind of moral relativism, but this only reinforces how great a challenge the diversity of moral opinion is for implementing moral knowledge in an AI system. Any such system will have to presume we already know things we do not know, even if the system merely aspires to auxiliary enhancement.

We have uncovered a deep dilemma facing the project of auxiliary enhancement. If the proposal is simply to create a system that assists users in making decisions that are more consistent and informed in light of their own prior values, then the degree of moral enhancement on offer is rather limited but pluralism is preserved. On the other hand, insofar as the system is preprogrammed to nudge users in the direction of this or that substantive ethical view, the system may collapse into the exhaustive approach, since the users who do not already have the ‘correct’ values will have to either do as the system tells them or continue in their immoral ways. Enhancement is only ‘auxiliary’ for those already on the path approved by the machine.

At least in the proposals laid out so far, AI moral enhancement proceeds as if we know things about morality we simply do not know. Van Wynsberghe and Robbins (2019, p. 730) capture one aspect of this problem succinctly thus: “Machines could only be better if there is some standard of moral truth with which to judge…If a machine was built which did somehow discover moral truths that have heretofore yet to be discovered (because morality would be a lot easier if we simply knew the moral truths) then one would have to accept on faith that machines are better than we are”. This is obvious for proposals that take up the exhaustive project, but the auxiliary project easily slips into the same conceptual difficulties. At least for the foreseeable future, giving and receiving moral advice will require some substantive and controversial judgments not easily reduced to the sorts of calculations or rule-governed inferences at which an AI might excel. It is time to consider enhancement that aims at cultivating those skills underlying practical wisdom. Instead of replacing or supplementing human judgment, such an approach to moral enhancement aims at improving human judgment. “The objective now would be for the user, with the exercise of their deliberative capacities, to learn to decide better and, with time, this would favour the ability to do so on one’s own” (Lara, 2021, p. 12).

## AI Socratic Interlocutor: Towards an AI that Knows It Does Not Know

Lara and Deckers propose an alternative auxiliary project that would engage the user in “the deliberative exchange in Socratic philosophy as an aid to develop better moral judgments” (2020, p. 281). They recognize that moral enhancement is something we must do each for ourselves in dialogue with others. Their proposal is to increase the users’ opportunities for that dialogue by training up a competent Socratic Interlocutor to serve as a discussion partner. While engaging humans in debate is challenging for today’s AI, it is plausible that such an AI could be developed in the near term, as IBM’s “Project Debater” demonstrates (Slonim et al., 2021). This goes beyond the one-sided attempts at ‘persuasion’ advocated by Klincewicz, since “the aim is to help the agent to learn to reason ethically, rather than to help the agent to learn which actions the system deems to be compatible with particular values” (Lara & Deckers, 2020, p. 282).

Their version of AI Socrates would be reduced to providing empirical support, conceptual clarity, argumentative logic, awareness of personal limitations, and advice on how to execute one’s decisions. However, since sound moral judgment demands more than pure logic and theory, they suggest the AI should also evaluate one’s judgment for ethical plausibility. This aspect of the system gestures towards substantive normative content, but it is offered as a neutrally presented knowledge of the history of debate in normative ethics. This insistence on neutrality is even more central to the proposal as it is developed in Lara (2021).

This moves the discussion in the right direction, but it still fails to model the full process of moral engagement as a socio-technical system. The system they propose is Socratic in some respects, but not in a rich way. The actual Socrates was not a neutral source of information and bland questioning to cover the logical space of inquiry; he was a flesh and blood agent with a point of view. He was a philosopher, not a neutral librarian. The Socratic Interlocutor proposed by Lara and Deckers embodies a minimalist moral perspective, but “a normative theory that any clever adolescent can apply, or that reaches practical conclusions that are in no way determined by premises about what is truly worth-while, serious, and so on, is guaranteed to be an inadequate theory” (Hursthouse, 1991, p. 232). Whatever advantages near-term AI might have over biased, emotional, rationally limited humans, we doubt they will be capable of nuanced judgment beyond that of any clever adolescent. This is decisive against the exhaustive approach, but it also has implications for the auxiliary project.

To address this problem, we propose a project of moral enhancement that more expansively models the concrete socio-technical system of moral progress thus far—viz., our ongoing philosophical conversation stretching back to the ancients, facilitated by books and other information technologies. Our conception of moral enhancement measures success just as we measure the success of any student of philosophy—i.e., in terms of the clarity, cogency, profundity, and sensitivity the student expresses in participating in the ongoing conversation. Genuine moral enhancement results from cultivating practical wisdom, and a well-trained AI interlocutor can contribute to such enhancement in just the ways upgrades to information and communications technologies (e.g., books) have helped to cultivate practical wisdom all these years past.

We do not have a morality ready-to-hand that we can program into a machine, and we must not overlook how the meaning of morality remains contested and negotiated in a community of agents thinking together about morality. To protect against the dangerous conflicts that might arise in this circumstance, we have evolved meta-virtues like tolerance and mutual respect to govern the ongoing debate and discussion, granting one another the space to explore particular hypotheses regarding the true, the beautiful, and the good as so many “experiments in living” (Mill, 1859, p. 54). If we mean to initiate a program of moral enhancement based on what we already know about morality, then we must model that process as well. Furthermore, since this process operates through a diversity of perspectives challenging one another, just as scientists each favor their own hypotheses while we count on the diverse scientific community to expose these biases, it will not do to abstract away from that diversity to create just one neutral moral machine. Rather, the total system needs to model the debate across perspectives as well. No competent philosopher would say, “Read this one book, and you’ll know all about morality”.

We need a diversity of machines debating with one another as well as with the user, since this models our actual moral experience. The total system we need to model is extended across minds connected by various information and communications technologies. What we are looking for in our quest for moral enhancement must be not a replacement for the humans in the system, but an upgrade to the technologies these humans use to discover and explore moral claims.

## Beyond a Logic Coach: The Total System as Constituted by a Diversity of Modules

To realize AI moral enhancement, we should model our actual process of moral engagement by having multiple AIs, each with their own points of view, in ongoing dialogue with one another. Other scholars already proposed AI systems based on Stoicism (Klincewicz, 2019) and Rawlsian principles of justice (Borinstein & Arkin, 2016). In our interpretation, each of them is offering a module, and there may even be competing modules from within a single wisdom tradition. The total system might include not only a virtual Socrates but also a virtual Epictetus, a virtual Confucius, … and more modern thinkers with competing or complementary visions to these. Each of these AI mentors would have a distinct point of view in ongoing dialogue with not only the user but also potentially with each other. The transcript of this dialogue would be like an ongoing and self-writing book of which the user is a co-author and ultimate publisher and editor—the user remains in charge of the process in the end. Pluralism is preserved, with each AI finding its meaning in that system as an upgrade to books and other information technologies, such that each is like a never-ending book and the total system is like a library of such books from across a multitude of diverse perspectives. While the total system approaches the liberal neutrality of the marketplace of ideas, as appropriate to the role of an excellent librarian, moral enhancement would come through direct engagement with the various perspectives in all their richness and complexity.

Since such a system is modular in nature, it would not have to be built all at once. This is crucial, given the enormity of the total project. Developing such modules represents a path to AI moral enhancement that approaches the limits of safety. Even books present certain opportunities for manipulation and disengagement of readers, but reading lots of books is generally regarded as a safe and effective method of moral improvement, especially when understood in terms of cultivating practical wisdom rather than compliance with specified outcomes.

Building such modules is not trivial, but it is plausibly within reach and may even be possible with technologies we already have. Already, systems like GPT-3 could be trained up, for example, on the complete works of Epictetus and all the commentaries on them and carry on a conversation that mostly works. Indeed, there is already a project training GPT-3 on the works of contemporary philosopher Daniel Dennett, which “creates textual outputs that Dennett experts often mistake for Dennett's own writing without parroting Dennett's exact words. It can synthesize *new* strings of Dennett-like prose” (Schwitzgebel, 2022, emphasis original, web; see also Schwitzgebel, Schwitzgebel, & Strasser, 2022). Other AI projects have resulted in systems that have already “discovered” how to count like a two-year-old (Alexander, 2019) and how to answer with some disturbing profundity questions about Life, the Universe, and Everything (Mueller, 2020). Whether such an interlocutor would be sufficient to exercise the faculties of students in a way leading to moral enhancement—measured in terms of the clarity, cogency, profundity, and sensitivity the student expresses in participating in the ongoing conversation— is an empirical matter. We do not need our books to be full-blown moral agents for them to succeed as tools in moral education; likewise, we do not need a useful AI tool for moral enhancement to be a full-blown moral agent. If the AI is good enough to engage the user in deeper practice in moral thinking, that may be enough. This is, after all, a large part of what ethicists already do in the classroom, and just having the conversation can be enough to accomplish moral enhancement along key dimensions (Jagger & Volkman, 2014). Such an AI just has to provoke meaningful engagement in the concrete way books and other information technologies already facilitate, while its interactivity promises an even richer engagement than books have provided heretofore.

## External vs. Internal Transcendence

It might be objected, especially by proponents of the exhaustive approach, that our proposal would not yield genuine moral enhancement. We do not *transcend* our human shortcomings by merely having on hand what are little more than interactive books, sophisticated though they may be. Indeed, our proposal does not take us very far beyond where we already are. But this is a feature, not a bug, since the aspiration to transcend our current moral situation is dangerous and maybe incoherent.

As Nussbaum (1990, p. 373) argues, there is a vision of moral transcendence that, like Calypso’s offer to make Odysseus a god, “begins to seem like the offer not so much of a better life, but of a different life, with different ends and excellences. And if one identifies oneself with the ends one already knows, one might well wonder whether one could in any meaningful sense survive the translation to such a life”. She urges us “to reject as incoherent…the aspiration to leave behind altogether the constitutive conditions of our humanity, and to seek for a life that is really the life of another sort of being—as if it were a higher and better life for *us*” (p. 379, emphasis original). This is not, however, to abandon all hope for moral transcendence. It is, after all, in the concrete process of moral engagement already embarked upon that we have articulated our many moral deficiencies. There is reason to hope we can continue to work out strategies for addressing these by cultivating practical wisdom and working out solutions grounded in our original starting place as concrete human agents.

The auxiliary approach that cultivates practical wisdom in ongoing dialogue with ‘AI mentors’ embodying distinct points of view promotes internal transcendence with minimal risk of sliding into the external transcendence of the exhaustive approach or those auxiliary approaches that share with it a conception of morality as reducible to the outputs of some algorithm. Ours is a much humbler approach to moral enhancement.

It might still be objected that the path to moral enhancement proposed here is every bit as arduous and liable to dead-end as the path we are presently on. It does nothing to simplify the process of moral improvement. If anything, by presenting the user with a bewildering array of competing perspectives, it is liable to make moral improvement *harder* rather than *easier*. We have scientists and technologists working to make things easier for us, but the proper role of the philosopher is to make things harder. By engaging with questions, we become skilled rather than deskilled in the ways of practical wisdom. Proposals for AI moral enhancement have generally tried to make things easier for us. But it is precisely the whole concrete socio-technical system of moral engagement—complete with the prospect that it may end in *aporia—*that we need to model if we are to pursue moral enhancement that does not presume to know things we do not already know. There are no shortcuts to moral excellence.

## Concluding Thoughts

We are content to call the interactive AI modules we have proposed ‘AI mentors’, even though we readily admit they are not *really* mentors. This is intended to convey the functional role these modules might play as dialogue partners, but they remain *tools*, not genuine mentors. While their robust interactivity is intended to enrich moral study and practice, thereby facilitating moral growth much as a Socratic discussion partner would, they play a role more analogous to books than to any human. Much as one should not confuse ‘Facebook friend’ for actual friend, one must not mistake an ‘AI mentor’ for an actual mentor.

The metaphor of mentoring intimates how these modules avoid many of the risks of more ambitious proposals for AI moral enhancement. An excellent mentor does not dictate what one shall do. To benefit from such a relationship, one must exercise one’s own judgment and develop one’s own practical wisdom. In contrast with other proposals, a system of AI mentors constitutes a library of the conversation in ethics that interactively facilitates engaging in that conversation but does not presume to know anything about morality that we do not already know. We submit this is a path to the sort of internal transcendence that constitutes genuine moral enhancement.

Although the project is ambitious, it is plausible such a system could be built in a piecemeal fashion with technologies already at hand or anticipated in the near future. In many ways, it is already a work in progress. Significantly, we could say much the same thing about morality itself.
